# Effects of dry fiber plus solubles and a carbohydrase complex on nutrient digestibility, ruminal fermentation, and performance of finishing cattle

**DOI:** 10.1093/tas/txag158

**Published:** 2026-10-27

**Authors:** Bárbara Martins Brixner, Daniel Montanher Polizel, Bruno Garcia de Carvalho Dias, Daiana dos Santos de Oliveira, Isaque Franciscato Vicci, João Pedro Galesi, Nicolas Ferreira Rodrigues, Larissa de Melo Coelho, Murillo Alves Porto Meschiatti, Diogo Fleury Azevedo Costa, Flávio Augusto Portela Santos

**Affiliations:** Animal Science Department, São Paulo University, Piracicaba, SP 13418–900, Brazil; Department of Biology and Animal Science, Sao Paulo State University, Ilha Solteira, SP 15385-000, Brazil; Animal Science Department, São Paulo University, Piracicaba, SP 13418–900, Brazil; Department of Animal Science, University of Nebraska, Lincoln, NE 68583-0908, United States; Animal Science Department, São Paulo University, Piracicaba, SP 13418–900, Brazil; Animal Science Department, São Paulo University, Piracicaba, SP 13418–900, Brazil; Department of Animal Science, University of Nebraska, Lincoln, NE 68583-0908, United States; Animal Science Department, São Paulo University, Piracicaba, SP 13418–900, Brazil; Animal Science Department, São Paulo University, Piracicaba, SP 13418–900, Brazil; Animal Science Department, São Paulo University, Piracicaba, SP 13418–900, Brazil; Center for Carbon Research in Tropical Agriculture (CCARBON), Luiz de Queiroz College of Agriculture (ESALQ), University of São Paulo (USP), Piracicaba, SP 13418-900, Brazil; Animal Science Department, São Paulo University, Piracicaba, SP 13418–900, Brazil; Animal Science Department, São Paulo University, Piracicaba, SP 13418–900, Brazil; Center for Carbon Research in Tropical Agriculture (CCARBON), Luiz de Queiroz College of Agriculture (ESALQ), University of São Paulo (USP), Piracicaba, SP 13418-900, Brazil; Institute for Future Farming Systems, CQUniversity, Rockhampton, QLD 4701, Australia; Animal Science Department, São Paulo University, Piracicaba, SP 13418–900, Brazil; Center for Carbon Research in Tropical Agriculture (CCARBON), Luiz de Queiroz College of Agriculture (ESALQ), University of São Paulo (USP), Piracicaba, SP 13418-900, Brazil

**Keywords:** β-glucanase, byproducts, enzymes, feed additives, xylanase

## Abstract

Two experiments were conducted to evaluate the effects of dry fiber plus solubles (**DFS**) inclusion, carbohydrase complex (**CC**) supplementation, and their interaction on nutrient digestibility, rumen fermentation parameters, performance and carcass characteristics of feedlot cattle. In Experiment 1, thirty rumen-cannulated Nellore steers were housed in 30 individual, fully covered pens with concrete floors, each equipped with an individual feed bunk and water. Steers were blocked by initial body weight (**BW = **264 ± 35.4 kg; standard deviation [**SD**]) and randomly assigned to 1 of 6 diets in a 2 × 3 factorial arrangement with inclusion of DFS (15 or 45% of diet dry matter [**DM**]) and CC levels (0, 75 or 100 mg/kg of diet DM). In Exp. 2, four hundred crossbred bulls (BW = 314 ± 40.2 kg; SD), were assigned to pens (*n* = 6 or 8 bulls/pen), and randomly assigned to receive the same 6 diets described in Exp. 1 (*n* = 10 pens/diet). The experiment lasted 123 d consisting of a 15 d adaptation (step-up) and 108 d in a finishing diet. In both studies, data were analyzed using PROC MIXED from SAS. Significance was declared at *P* < 0.05. In Exp. 1, the inclusion of 45% of DFS increased neutral detergent fiber (**NDF**), ether extract (**EE**) and ash intake (*P* < 0.05), with no effect on dry matter intake (**DMI**) and nutrient digestibility (*P* > 0.05). CC did not affect nutrient intake (*P* > 0.05), but increased EE digestibility (*P* = 0.02). There was DFS × CC interaction for crude protein (**CP**) digestibility (*P* < 0.05), which increased with doses of CC in the diet containing 45% DFS. There were no interactions (*P* > 0.05) between DFS and CC doses for rumen fermentation parameters. The inclusion of 45% DFS increased ruminal pH (*P* = 0.03), with no effect on rumen short-chain fatty acids (**SCFA**) profile (*P* > 0.05). The CC decreased rumen acetate (*P* = 0.02), increased propionate (*P* = 0.01), and, consequently, decreased acetate:propionate ratio (**Ac:Prop ratio**) (*P* = 0.01). In Exp. 2, there was no DFS and CC doses interaction (*P* > 0.05). The inclusion of 45% DFS in the diet increased average daily gain (**ADG**) (*P* = 0.02), feed efficiency (**G:F**) (*P* = 0.02), net energy for maintenance (**NEm**) (*P* = 0.03) and net energy for gain (**NEg)** (*P* = 0.04) and decreased the content of fecal starch (*P* < 0.0001) compared with the inclusion of 15%. CC did not affect the performance and carcass characteristics of feedlot cattle (*P* > 0.05). In conclusion, the higher inclusion of DFS improved animal performance, whereas the CC modified ruminal fermentation patterns by increasing the molar proportion of propionate, with no effects on performance.

## Teaser

Carbohydrase complex supplementation shifted ruminal fermentation toward greater propionate production without improving feedlot performance. Dry fiber plus solubles increase the rumen pH and improve average daily gain and feed efficiency of feedlot cattle.

## Introduction

Diets with high energy density are commonly used in feedlot systems to increase weight gain and feed efficiency, as well as to improve carcass characteristics (Crawford et al. 2022; Chen et al. 2024). In these systems, corn is the primary cereal grain used in feedlot cattle diets in the United States and Brazil (Samuelson et al. 2016; Monsalve and Millen 2025). However, by-products from the corn ethanol industry have increasingly been incorporated into feedlot diets due to their competitive cost and favorable nutritional profile. Among these by-products, dried distillers grains with solubles (**DDGS**) are widely used and are characterized by containing approximately 30.8% crude protein (**CP**), 33.7% neutral detergent fiber (**NDF**), and 3.2 Mcal/kg of metabolizable energy (**ME**) (NASEM 2016). More recently, advances in ethanol production technology, particularly the separation of fiber prior to fermentation, have resulted in new co-products such as wet and dry corn bran with distillers solubles (Antunes et al. 2025). These ingredients typically contain greater fiber concentrations and moderate protein levels, which may influence their feeding value in high-concentrate diets. For example, Lima et al. (2022) evaluated reduced-fat wet fiber plus solubles (**WFS**), containing 33.9% CP, 6.79% ether extract (**EE**), and 52.5% NDF, in finishing cattle diets based on sugarcane bagasse, ground flint corn, pelleted citrus pulp, soybean meal, urea, and a mineral–vitamin premix. The inclusion of WFS at 45% of diet DM, replacing a mixture of ground flint corn, citrus pulp, soybean meal, and urea, reduced dry matter intake (**DMI**) by 12%, tended to increase carcass-adjusted feed efficiency (**G:F**) by 16%, and increased observed dietary net energy for maintenance (**NEm**) by 9.85%. These results suggest that WFS may represent an energetically competitive ingredient in finishing diets; however, further evaluation is needed to better understand its feeding value and its effects on animal performance when included at high levels in feedlot diets. Because ingredients such as WFS and dry fiber plus solubles (**DFS**) contain substantial amounts of fibrous carbohydrates, strategies to improve the utilization of these components have also been investigated. Among these strategies, the use of feed additives, such as enzyme complexes, has been proposed to enhance the degradation of proteins, fiber, and nonfiber carbohydrates (**NFC**), with the choice of enzyme depending on the type and composition of the diet (Beauchemin et al. 2004). Enzymes capable of degrading carbohydrates (carbohydrase complex; **CC**) are used to increase the utilization of less digestible components, such as the fibrous fraction, facilitating cell wall degradation and improving access to nutrients within plant cells. Studies have shown that supplementation with enzymes such as xylanase, β-glucanase, and arabinofuranosidase can improve energy and protein utilization and increase nutrient digestibility in poultry diets, resulting in improved performance (Gilani et al. 2021; McCafferty et al. 2022; Anwar et al. 2023; Fortes et al. 2024). Nevertheless, the results of enzyme supplementation for ruminants are controversial (Ferreira et al. 2025), being often influenced by dosage, diet composition (for example, roughage: concentrate ratio), the stability of the enzyme and the method of application in the diet (Beauchemin et al. 2004; Sujani and Seresinhe 2015; Lago et al. 2025).

Therefore, the aim of this study was to evaluate the digestibility of nutrients, ruminal fermentation characteristics, performance and carcass characteristics of feedlot cattle, receiving diets with high or low inclusion of DFS and CC levels. The DFS inclusion levels aimed to result in diets containing different concentrations of NFC and NDF, allowing the factorial arrangement of treatments (2 × 3) to allow evaluating the interaction of this nutritional variation in the diets, caused by DFS, with the CC levels. The hypothesis of the present study was that the inclusion of higher levels of DFS would improve efficiency of ruminal fermentation, ruminal pH, diet energy content and cattle performance. Furthermore, the use of CC could increase fiber digestibility, especially in diets containing higher concentrations of DFS, resulting in increased animal performance.

## Materials and methods

Two experiments were conducted at the facility of the Animal Science Department at the University of São Paulo (USP), Escola Superior de Agricultura Luiz de Queiroz (ESALQ), located in Piracicaba, São Paulo, Brazil (22°43′31ʺ S, 47°38′51ʺ W, and elevation of 546 m). All animals utilized herein were cared for in accordance with acceptable practices and experimental protocols reviewed and approved by the ESALQ/USP Institutional Animal Care and Use Committee (#3701051020).

### Experiment 1: Metabolism

#### Animals, housing, and diets

Thirty rumen-cannulated Nellore (*Bos indicus*) steers [initial body weight (**BW**) = 264 ± 35.4 kg; age = 25 ± 2 mo] were assigned to individual pens, with concrete surface, feed bunk and waterer, in a randomized complete block design according to their initial shrunk BW. Within blocks (*n* = 5), steers were randomly assigned to 1 of 6 experimental diets defined by a 2 × 3 factorial arrangement in which: factor 1 was determined by the inclusion of 15 or 45% of DFS (dry matter; **DM** basis; FS Bioenergia, Lucas do Rio Verde, MT, Brazil) while factor 2 was defined by the inclusion of 0, 75, and 100 mg of CC/kg of diet DM. The DFS used was composed of 88.2% of DM, 7.1% of ash, 22.8% of CP, 42.2% of NDF, and 6.6% of ether extract (**EE**). The CC used in the present study was composed of 40.8% β-glucanases and 59.2% xylanases (Adisseo Company, São Paulo, São Paulo, Brazil). The enzyme complex is primarily characterized by xylanase activity, which represents its main enzymatic function. The product is obtained through industrial fermentation of *Talaromyces versatilis* (IMI378536 and DSM26702; Adisseo France S.A.S. proprietary strains) which were previously homogenized in the mineral premix to ensure a more uniform distribution in the diet. According to product specifications, the enzyme provided 64.5 UV/g of β-glucanase and 93.7 UV/g of xylanase activity, with UV corresponding to the activity unit defined by the supplier. The enzyme inclusion rate was calculated based on daily dry matter intake to achieve the targeted enzymatic activity per kilogram of dietary DM. Diets were prepared daily to minimize potential loss of enzymatic activity.

The steers were already adapted to high-concentrate diets before the beginning of the experiment. The experiment lasted 24 d, with the first 17 d used primarily for adaptation to the experimental diets, and the last 7 d for data collection.

Throughout the experimental period, the steers received diets containing 8% of sugar cane bagasse and 92% of concentrate. The sugar cane bagasse average particle length distribution was 13% > 19 mm; 52% < 19 mm and > 8 mm; and 35% < 8 mm (bottom sieve). Corn was processed through a hammer mill to achieve a mean particle size of 2.21 mm (Table 1), according to procedures described by Yu et al. (1998), using sieves with 6.0, 3.5, 2.0, and 1.25-mm square pores (EAGRI Equipamentos Agrícolas, Dourados, MS, Brazil). The experimental diets were prepared using a horizontal mixer with a capacity of 1500 kg (Casale Rotormix Express 4.00, Sao Carlos, SP, Brazil), with a precision of 1 kg and a tipping mixing system. The corn, DFS, and sugarcane bagasse were weighed in the mixer, while the urea and mineral premix were previously weighed on a scale with a capacity of 100 kg, with a precision of 0.1 kg (Marte Marte Cientifica, Sao Paulo, SP, Brazil) and placed in the mixer and the mixing time for all diets was 5 min. Between the preparation of each of the 6 diets, the mixer was cleaned so that there was no contamination between treatments. The feed offered was calculated daily based on the previous day individual DMI. The experimental diets were offered once daily at 0600 h. During the collection period, to reduce the amount of orts, daily feed intake was restricted to 90% of the average feed intake of that specific steer measured during the last 5 days of the adaptation period as described by Zinn (1990).

**Table 1:** Ground corn particle size distribution for Exp. 1 and 2.

Pores in the sieve	% of total
**> 6.00 mm**	0.00
**< 6.00 and > 3.50 mm**	0.60
**< 3.50 and > 2.00 mm**	55.2
**< 2.00 and > 1.25 mm**	39.5
**< 1.25 mm**	4.7
**Mean particle size of corn, mm^a^**	2.21

a Corn retained on the 6 mm screen was determined in 20 randomly selected particles using a digital caliper. The residue retained in the bottom was assumed to have a mean particle size of 0.625 mm. Based on Yu et al. (1998).

#### Body weight measurements and collections

The steers were weighed at the beginning and end of the experiment after 16 h of feed and water withdrawal, in which the initial BW was used to perform the randomization of the animals into blocks and treatments. The feed offer from all treatments and ingredients was sampled from day 18 to 24, stored at −18 °C for subsequently DM analysis.

From d 18 to 22, total fecal production was individually collected, weighed, sampled (approximately 10% of wet weight), and frozen at −18 °C for DM analyses and determination of total fecal excretion. The pens were monitored every 3 h of each day to check if there was the presence of feces. Pens used to allocate the animals in this experiment were built with a 1% slope to separate and minimize urine contamination in the feces sample (Soares et al. 2023). From d 23 to 24, approximately 100 g of fecal samples were collected directly from the rectum of each animal, every 3 h, starting immediately before feeding (0 h), and stored at −18 °C for nutrient analysis and subsequent apparent nutrient digestibility calculations (Dias et al. 2022). These collections were staggered over sampling hours, in a manner that all collections represented one full day. Specifically, on day 23 samples were collected at hours 0, 6, 12 and 18, and on day 24 were collected at hours 3, 9, 15 and 21. Apparent digestibility was calculated according to the formula: TTAD (%) = (((DMI × NCDM) – (FDM × NCFM)) × 100)/(DMI × NCDM), where TTAD = total tract apparent digestibility, DMI = dry matter intake, NCDM = nutrient content of the DMI (%), FDM = fecal dry matter, and NCFM =nutrient content of the fecal DM (%).

The rumen fluid was collected on days 23 and 24 of the experimental period at the same hours of fecal collection from the rectum. A representative sample of ruminal content was collected from three different regions (caudal, ventral, and cranial sac) of each animal, which was filtered with a nylon cloth to obtain approximately 100 mL of ruminal fluid. The solid phases of the ruminal content were returned to the rumen, and the fluid was used to determine the pH using a potentiometer (DIGIMED DM20, Sao Paulo, Brazil). Two aliquots were prepared and stored at −18 °C for subsequent analysis of short-chain fatty acids (**SCFA**; molar proportion of each SCFA, total SCFA), acetate:propionate ratio (**Ac:Prop ratio**) and rumen ammonia.

#### Sample analysis

Samples of feed offer and feces were thawed, dried in a forced-air oven at 55 °C for 72 h, and ground through a 1-mm Wiley Mill screen (Marconi, Piracicaba, SP, Brazil). The final DM content was determined after drying in an oven at 105 °C for 24 h (AOAC 1990; method #934.01). Ash concentration was determined by incinerating the samples in a muffle furnace at 550 °C for 4 h (AOAC 1990; method #942.05). The EE was determined according to AOAC (1990; method #920.85). The NDF was performed according to Van Soest et al. (1991) in an Ankom 2000 fiber analyzer (Ankom Tech. Corp., Macedon, NY, USA), using sodium sulfite and heat-stable α-amylase. Total nitrogen concentration was determined using a LECO TruMac N Analyzer (Leco Corporation, St. Joseph, MI; AOAC 1990; method #968.06). CP was calculated by multiplying the total N content by 6.25. Total carbohydrates (**TCHO)** were calculated as: TCHO (%) = 100 − [CP (%) + EE (%) + ash (%)]. NFC (%) was calculated as: NFC = 100 − (CP, % + NDF, % + Ash, % + EE, %), according to Mertens (1997). The total digestible nutrients (**TDN**) values for each diet were estimated based on the digestibility data as:

TDN = [kg of DCP + kg of DTCHO + (kg of DEE × 2.25)]/kg of DMI (Weiss et al. 1992; NRC 2001).

Ruminal fluid samples were thawed at room temperature, and 2.0-mL aliquots were transferred to microtubes and centrifuged at 15,000 × *g* for 60 min at 4 °C. The supernatant was used to analyze the SCFA and rumen ammonia concentration according to procedures described by Ferreira et al. (2016) and Broderick and Kang (1980), respectively.

### Experiment 2: Performance

#### Pre-trial period, health protocol, housing, and diets

Prior to the start of the experiment, bulls grazed tropical pastures and received a protein-energy supplement. On arrival, the animals were individually identified with ear tags, vaccinated against clostridial pathogens (Resguard Multi-Biovet), following the dosage recommended by the company of 5 mL and dewormed (Ricobendazole 13.6% 500 mL—Chemitec) at a dosage of 1.0 mL/40 kg of BW. From d −30 to −1 of the study, all animals were housed in group-pens with ad libitum access to water and a diet containing 30% of sugar cane bagasse and 70% of concentrate (DM basis).

#### Experimental animals, statistical design, adaptation period and final diet

A total of 400 Red Brangus, Braford and F1 Red Angus × Nellore bulls were initially enrolled in the experiment. Eleven animals were subsequently removed because of health disorders unrelated to the experimental treatments; therefore, data from 389 bulls were included in the final statistical analysis. On d 0 of the experiment, bulls were weighed after 16 h of feed and water withdrawal (initial BW = 314 ± 40.2 kg), and shrunk BW was used to assign animals to treatments in a randomized complete block design. The 60 pens were arranged in 10 complete blocks, with 6 pens per block, and within each block, pens were randomly assigned to 1 of 6 experimental diets. The experimental diets were defined by a 2 × 3 factorial arrangement, in which factor 1 was DFS inclusion (15 or 45% of diet DM) and factor 2 was CC levels (0, 75, or 100 mg/kg of diet DM). Bulls were housed in group pens with concrete floors and partial roofing, each equipped with a concrete feed bunk and a waterer shared between two adjacent pens. The experiment lasted 123 d, including a 15 d step-up adaptation period using three transition diets (30% forage for 5 d, 20% forage for 5 d, and 10% forage for 5 d), followed by the finishing period. Experimental treatments (DFS inclusion and CC levels) were provided from the first day of the adaptation period. The experimental diets (Table 2) were formulated to meet the nutritional requirements of the animals, with an average gain of around 1.5 kg/d, according to NASEM (2016) and contained 30 ppm of sodium monensin (Rumensin, ELANCO Animal and Health). The DM of the diets was set at 70%, with adjustment made through the inclusion of water in variable amounts depending on the DM content of the ingredients, mainly sugarcane bagasse, which was evaluated every two days.

**Table 2:** Nutritional profile of the diets offered during Exp. 1 and 2.

Items	Diets^a^							
	Adap—1		Adap—2		Adap—3		Final	
	15% DFS	45% DFS	15% DFS	45% DFS	15% DFS	45% DFS	15% DFS	45% DFS
**Inclusion, % DM**								
** Sugar cane bagasse**	30.0	30.0	20.0	20.0	10.0	10.0	8.50	8.50
** Ground corn**	52.0	23.0	62.0	33.0	72.0	43.0	73.0	44.0
** DFS^b^**	15.0	45.0	15.0	45.0	15.0	45.0	15.0	45.0
** Urea**	1.00	0.00	1.00	0.00	1.00	0.00	1.50	0.50
** Mineral premix^c^^,^^d^**	2.00	2.00	2.00	2.00	2.00	2.00	2.00	2.00
**Nutritional profile^e^**								
** DM**	75.5	75.9	79.4	79.4	83.3	83.6	84.2	84.4
** OM**	93.0	91.4	93.8	92.2	94.5	93.0	94.6	92.7
** CP**	12.1	13.2	12.4	13.8	13.1	14.4	14.0	15.5
** NDF**	31.3	41.9	24.9	35.5	18.5	29.2	17.6	28.2
** EE**	4.63	4.70	5.22	5.29	5.81	5.88	5.87	5.93
** Total CHO**	75.3	73.5	76.1	73.1	75.6	72.7	76.8	75.3
** NFC**	45.0	31.6	51.3	37.6	57.1	43.5	59.3	47.1

a Adap—1, adaptation diet 1; Adap—2, adaptation diet 2; Adap—3, adaptation diet 3; Final, final diet.

b DFS, dry fiber plus solubles.

c Composition: 160 g/kg Ca, 20 g/kg Mg, 15 g/kg Na, 80 g/kg K, 330 mg/kg Cu, 1300 mg/kg Mn, 3000 mg/kg Zn, 16.6 mg/kg Co, 30 mg/kg I, 5 mg/kg Se, 65,000 IU Vit. A, 14,000 IU Vit. D3, 500 IU Vit. E, 1500 mg/kg of monensin.

d The carbohydrase complex was included directly in the mineral premix, at doses of 0.00 (without inclusion), 75 and 100 mg/kg of DM.

e DM, dry matter. OM, organic matter; CP, crude protein; NDF, neutral detergent fiber; EE, ether extract; Total CHO, total carbohydrates; NFC, nonfiber carbohydrates.

Throughout the experiment, the diets were offered once daily as a total mixed ration using a feed wagon (Rotormix-40; Casale Equipamentos, Sao Carlos, SP, Brazil) with an electronic scale. The preparation of the experimental diets was carried out as described in Exp. 1, and the total mixed ration (**TMR**) was offered to ensure ad libitum intake and result in 3% orts. Orts were removed and weighed three times weekly, and samples were dried in a forced-air oven at 105 °C for 24 h to determine dry matter (AOAC 1990; #934.01).

#### Body weight measurements and feeds and orts, and fecal collections

Individual shrunk BW was collected after 16 h of feed and water withdrawal on d 0 and 123 of the experimental period, and the average daily gain (**ADG**) was calculated. The DMI was evaluated daily throughout the experiment by weighing the feed offered and orts in the following day. At the end of the experiment, the ADG and DMI were used to determine the G:F.

On days 30, 60, and 90 of the experiment, fecal samples were collected directly from the concrete floor from 3 animals per pen, immediately after defecation at approximately 0700 h, taking care to collect only the upper portion of the feces to avoid contamination with manure from the pen. The samples were placed in plastic bags and immediately packed in styrofoam with ice, in order to avoid microbial activity, and subsequently stored at −20 °C in a freezer. For the analysis, samples from each day of collection were thawed, composed per pen (one sample per pen per day of collection), dried in a forced air oven at 55 °C for 72 h and ground using a “Wiley” type mill (MA -680 Marconi Ltda, Piracicaba, SP, Brazil) through a 1-mm sieve. Analysis was performed using a Total Starch Assay Kit (K-TSTA; Megazyme, Chicago, IL) according to AOAC (2005); 996.11 and AACC (2010); # 76-13.01.

Samples of dietary ingredients were collected weekly and pooled over the experimental period to obtain representative composite samples, which were analyzed for chemical composition (Table 2). Samples of each experimental diet were also collected weekly throughout the experiment. At the end of the experimental period, weekly samples from each diet were pooled to obtain one representative composite sample per diet, resulting in six composite diet samples. The composite samples were analyzed for chemical composition (ESALQLab; Piracicaba, SP, Brazil) in duplicate using the same procedures described in Exp. 1.

The energy calculation was based on the average data of the pens for the variables of BW, DMI and ADG. The observed values of net energy of the rations were calculated according to the methodology proposed by Zinn and Shen (1998). The energy gain (**EG**) was calculated using the equation EG = (0.0557 × BW^0.75^) × ADG^1.097^ (NRC 1984), in which EG is daily energy deposited (Mcal/d) and BW is mean shrunk BW. The equation used to determine the maintenance energy expended (**MEx**; Mcal/d) was MEx = 0.077 × BW^0.75^ (NRC 1996). From the calculated amounts of NEm and energy required for gain (**NEg**), the NEm of each diet was obtained by the quadratic equation NEm = [–b ± (b^2^ − 4ac)^1/2^]/2a, in which a = −0.877 × DMI, b = 0.877 × MEx + 0.41 × DMI + EG, and c = −0.42 × MEx and NEg of each diet was obtained by the equation NEg = 0.877 × NEm – 0.41 (Zinn and Shen 1998).

On d 123, all bulls were subjected to ultrasound evaluations (Aloka SSD-500V with a 17.2 cm/3.50 MHz convex probe; Hitachi Healthcare Americas, Twinsburg, OH), according to procedures described by the Ultrasound Guidelines Council (UGC 2014). Through this evaluation, ribeye area (**REA**) and backfat thickness (**BFT**) of the *Longissimus thoracis* muscle between the 12^th^ and 13^th^ ribs were measured.

After the final weighing (d 123), the animals were sent to the commercial packing plant in Sertãozinho/SP. After slaughter, hot carcass weight (**HCW**) was determined and the dressing percentage (**DP**) was obtained by the equation DP = (HCW/final BW) × 100.

### Statistical analysis

For both experiments, the data were analyzed using the PROC MIXED of SAS (Version 9.0; SAS Inst. Inc. Cary, NC). The data were analyzed for normality of residues, homogeneity of variances, and removal of outliers. The Kenward-Roger approximation was used to determine the denominator degrees of freedom for tests of fixed effects. Block was included as a random effect. Data were analyzed as a 2 × 3 factorial arrangement of treatments, with DFS inclusion and CC level as fixed effects, as well as their interaction. Significance was declared at *P* < 0.05. When a significant effect of CC level was detected, linear (*L*) and quadratic (*Q*) orthogonal polynomial contrasts were evaluated.

Exp. 1. The animal was considered the experimental unit. Rumen fermentation variables (SCFA, pH, and ammonia) were analyzed as repeated measures over time, with fixed effects of DFS inclusion, CC level, DFS inclusion × CC level, hour, and their resulting interactions. Animal was included as the random effect. A first-order autoregressive covariance structure was used based on the lowest Akaike Information Criterion (AIC). For nutrient intake and digestibility, the model included the fixed effects of DFS inclusion, CC level, and their interaction.

Exp. 2. The pen was considered the experimental unit. The statistical model for BW, ADG, DMI, G:F, and carcass characteristics included the fixed effects of DFS inclusion, CC level, and their interaction, with block included as a random effect. Fecal starch concentration was analyzed as repeated measures over time, with fixed effects of DFS inclusion, CC level, DFS inclusion × CC level, day, and their resulting interactions. A first-order autoregressive covariance structure was selected based on the lowest AIC.

## Results

### Experiment 1: Metabolism

No DFS × CC interaction was observed for nutrient intake (*P* ≥ 0.59; Table 3). DFS inclusion did not affect the DM, organic matter (**OM**), CP, TCHO, NFC and TDN intake (*P* ≥ 0.11); however, increased the NDF (*P* = 0.01) and EE intake (*P* < 0.01). CC levels did not affect the DM or nutrients intake (*P* ≥ 0.19).

**Table 3:** Nutrient intake and digestibility in nellore steers receiving high-concentrate diets containing 15 or 45% of DFS and increasing CC levels (0, 75, and 100 mg/kg of DM).

Items^b^	DFS^a^		CC^a^			SEM	*P-*value		
	15%	45%	0	75	100		DFS	CC	DFS × CC
**Intake, kg/d**									
** DM**	4.87	5.43	4.82	5.21	5.42	0.38	0.26	0.60	0.83
** OM**	4.41	4.81	4.24	4.71	4.88	0.33	0.38	0.48	0.80
** CP**	0.60	0.71	0.61	0.67	0.69	0.06	0.11	0.56	0.59
** NDF**	1.12	1.55	1.26	1.34	1.39	0.12	0.01	0.73	0.74
** EE**	0.21	0.30	0.22	0.27	0.28	0.03	<0.01	0.19	0.68
** Total CHO**	3.57	3.76	3.37	3.74	3.87	0.25	0.60	0.50	0.84
** NFC**	2.45	2.21	2.12	2.39	2.48	0.18	0.28	0.38	0.89
** TDN**	3.90	4.29	3.74	4.22	4.33	0.39	0.38	0.52	0.76
**Total tract apparent digestibility, %**									
** DM**	84.2	83.0	83.3	83.1	84.3	1.21	0.42	0.78	0.13
** OM**	84.4	82.9	83.1	83.3	84.5	1.21	0.31	0.69	0.11
** CP**	76.5	76.9	74.7	76.6	78.8	2.30	0.86	0.38	0.05
** NDF**	70.1	68.4	68.4	68.0	71.4	2.79	0.62	0.67	0.25
** EE**	90.8	90.1	88.25	90.6	92.5	1.39	0.57	0.02	0.08
** Total CHO**	85.2	83.4	84.1	83.9	84.9	1.20	0.20	0.28	0.17
** NFC**	91.5	92.2	92.0	91.7	91.9	0.73	0.46	0.94	0.24
** TDN**	81.0	78.5	77.3	80.3	81.6	1.45	0.12	0.09	0.10

a DFS, dry fiber plus solubles; CC, carbohydrase complex. The CC was included at 0 (no inclusion), 75, and 100 mg/kg of diet DM. Effects of DFS, CC, and their interaction (DFS × CC) are presented. Significance was declared at *P* < 0.05.

b DM, dry matter. OM, organic matter; CP, crude protein; NDF, neutral detergent fiber; EE, ether extract; Total CHO, total carbohydrates; NFC, nonfiber carbohydrates; TDN, total digestible nutrients.

There was an interaction between DFS inclusion and CC levels for CP digestibility (*P* = 0.05; Table 3), with no interaction observed for the digestibility of the other nutrients (*P* ≥ 0.08). CC inclusion linearly increased CP digestibility (*P* = 0.049) in diets containing 45% DFS; however, there was no linear effect of CC levels in diets containing 15% DFS (*P* = 0.48; Fig. 1). CC levels affected EE digestibility (*P* = 0.02), which increased linearly with increasing CC inclusion (*P* = 0.01). There was no effect of CC levels on the digestibility of the other nutrients (*P* ≥ 0.09). DFS inclusion did not affect DM digestibility or the digestibility of nutrients other than CP (*P* ≥ 0.12).

**Figure 1:**
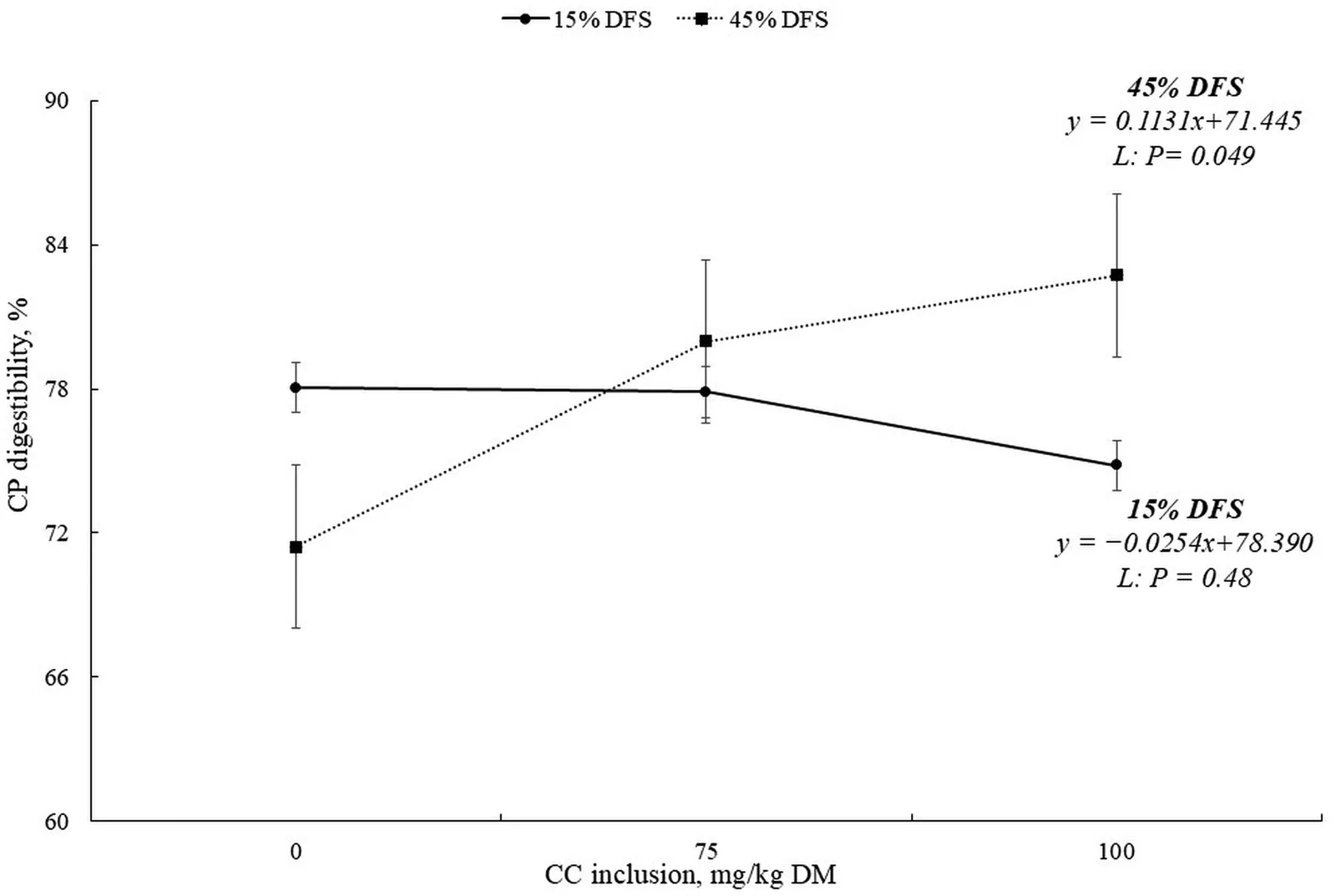
Crude protein (CP) digestibility in diets containing 15 or 45% dry fiber plus solubles (DFS) according to carbohydrase complex (CC) inclusion (0, 75, or 100 mg/kg). points represent least squares means and error bars represent the standard error of the mean. Increasing CC inclusion linearly increased CP digestibility in diets containing 45% DFS (*P* = 0.049), whereas no linear effect was observed in diets containing 15% DFS (*P* = 0.48). regression equations for each DFS inclusion level are presented in the figure.

No interaction between DFS inclusion and CC levels was observed for rumen fermentation variables (*P* ≥ 0.12). DFS inclusion did not affect SCFA molar proportions, Ac:Prop ratio, AcBut: Prop, or ammonia nitrogen (*P* ≥ 0.34). No interaction was observed between experimental treatments and sampling time (*P* ≥ 0.10) for rumen fermentation variables (Table 4). Sampling time affected all variables (*P* < 0.01), except valerate molar proportion (*P* = 0.43). However, inclusion of 45% DFS increased ruminal pH (*P* = 0.03). CC levels affected the molar proportions of acetate (*P* = 0.03) and propionate (*P* = 0.03), as well as the Ac:Prop ratio (*P* = 0.03) and AcBut:Prop ratio (*P* = 0.03). Orthogonal polynomial contrasts indicated that increasing CC inclusion linearly increased propionate (*P* = 0.01) and linearly decreased acetate (*P* = 0.02), Ac:Prop ratio (*P* = 0.01), and AcBut:Prop ratio (*P* = 0.01). There was no effect of CC levels on the other fermentation variables (*P* ≥ 0.13).

**Table 4:** Rumen short-chain fatty acids, pH, and ammonia concentration of *B. indicus* steers receiving high-concentrate diets containing 15 or 45% of DFS and increasing levels of carbohydrase complex (0, 75, and 100 mg/kg of DM).

Items^b^	DFS^a^		CC^a^			SEM	*P*-value						
	15%	45%	0	75	100		DFS	CC	DFS × CC	Hour	DFS × Hour	CC × Hour	DFS × CC ×Hour
**SCFA, mol/100 mol**													
** Acetate**	55.10	54.71	56.95	53.58	54.19	0.90	0.72	0.03	0.66	<0.01	0.11	0.47	0.49
** Propionate**	29.47	29.66	26.62	31.01	31.09	1.25	0.90	0.03	0.71	<0.01	0.54	0.65	0.54
** Isobutyrate**	1.01	1.02	1.06	1.02	0.97	0.07	0.91	0.57	0.89	<0.01	0.92	0.81	0.77
** Butyrate**	10.43	10.10	10.83	7.12	9.85	0.41	0.50	0.24	0.16	<0.01	0.62	0.32	0.60
** Isovalerate**	2.05	2.01	2.25	2.06	1.78	0.19	0.84	0.25	0.64	<0.01	0.87	0.71	0.10
** Valerate**	1.18	1.16	1.17	1.19	1.15	0.03	0.58	0.75	0.25	0.43	0.81	0.78	0.99
** Total SCFA, mM**	71.78	68.10	66.85	73.84	69.13	3.37	0.34	0.32	0.58	<0.01	0.32	0.86	0.42
** Ac:Prop**	2.02	1.97	2.26	1.86	1.86	0.12	0.76	0.03	0.84	<0.01	0.56	0.76	0.56
** AcBut:Prop**	2.41	2.34	2.70	2.22	2.22	0.14	0.69	0.03	0.76	<0.01	0.75	0.77	0.37
** Ammonia, mg/dL**	11.33	11.39	11.74	10.28	12.05	0.64	0.94	0.13	0.12	<0.01	0.42	0.35	0.18
** Rumen pH**	6.24	6.40	6.37	6.35	6.24	0.06	0.03	0.28	0.57	<0.01	0.10	0.40	0.87

a DFS, dry fiber plus solubles; CC, carbohydrase complex; Hour = sampling time. Effects of DFS, CC, Hour, and their interactions (DFS × CC, DFS × Hour, CC × Hour, and DFS × CC × Hour) are presented. Significance was declared at *P* < 0.05.

b SCFA, short-chain fatty acids; Ac, Prop, acetate:propionate ratio; AcBut, Prop ratio, (acetate + butyrate):propionate ratio.

### Experiment 2: Performance

No interaction between DFS inclusion and CC levels was observed for final BW, ADG, DMI (kg/d and % BW), or G:F (*P* ≥ 0.54; Table 5). At the beginning of the experiment, BW did not differ among treatments (*P* ≥ 0.99; Table 5). Inclusion of 45% DFS increased ADG (*P* = 0.02) compared with inclusion of 15% DFS; however, there was no effect on final BW (*P* = 0.34). DFS inclusion did not affect DMI, expressed as kg/d (*P* = 0.45) or % BW (*P* = 0.96), but increased G:F when 45% DFS was included in the diet (*P* = 0.02). CC inclusion did not affect final BW, ADG, DMI (kg/d and % BW), or G:F (*P* ≥ 0.53). There was no effect of DFS inclusion, CC levels, or their interaction on hot carcass weight, dressing percentage, longissimus muscle area, or subcutaneous fat thickness (*P* ≥ 0.14).

**Table 5:** Feedlot performance and carcass characteristics of crossbred feedlot bulls receiving high-concentrate diets containing 15 or 45% of DFS and increasing CC levels (0, 75, and 100 mg/kg of DM).

Items^b^	DFS^a^		CC^a^			SEM	*P*-value		
	15%	45%	0	75	100		DFS	CC	DFS × CC
**Performance data**									
** BW, kg**									
** Initial**	314.1	314.1	314.2	314.1	314.0	9.12	0.99	0.99	0.99
** Final**	471.3	482.7	477.2	476.0	477.7	10.17	0.34	0.99	0.93
** ADG, kg**	1.28	1.37	1.33	1.31	1.33	0.03	0.02	0.92	0.56
** DMI, kg/d**	8.83	8.99	8.98	8.77	8.97	0.18	0.45	0.66	0.62
** DMI, %BW**	2.26	2.26	2.28	2.23	2.27	0.03	0.96	0.53	0.54
** G:F**	0.145	0.154	0.149	0.151	0.149	<0.01	0.02	0.93	0.75
**Carcass data**									
** Hot carcass weight, kg**	262.5	270.3	266.4	265.9	267.0	6.19	0.28	0.99	0.89
** Dressing percent, %**	55.78	55.95	55.89	55.84	55.88	0.22	0.51	0.98	0.63
** *Longissimus* muscle area, cm^2^**	81.3	83.5	83.0	81.7	82.4	1.25	0.14	0.76	0.52
** Subcutaneous fat thickness, mm**	6.35	6.54	6.66	6.32	6.36	0.25	0.51	0.59	0.32

a DFS, dry fiber plus solubles; CC, carbohydrase complex. The CC was included at 0 (no inclusion), 75, and 100 mg/kg of diet DM. Effects of DFS, CC, and their interaction (DFS × CC) are presented. Significance was declared at *P* < 0.05.

b BW, body weight; ADG, average daily gain; DMI, dry matter intake; G:F, gain-to-feed (feed efficiency).

No interaction was observed between DFS, CC levels and day for fecal starch concentration (*P* = 0.09). The CC levels and day interaction was observed (*P* = 0.03; Fig. 2) in which on days 30 and 60 of the study the inclusion of CC levels did not affect the concentration of fecal starch, however, on day 90 there was a decreasing linear effect for the concentration of fecal starch with the inclusion of CC levels. In addition, there was no DFS and day interaction (*P* = 0.68), but an effect of the inclusion of DFS was observed (*P* < 0.01; Fig. 3) in which the inclusion of 45% decreased the concentration of fecal starch compared to the diet containing 15% of DFS.

**Figure 2:**
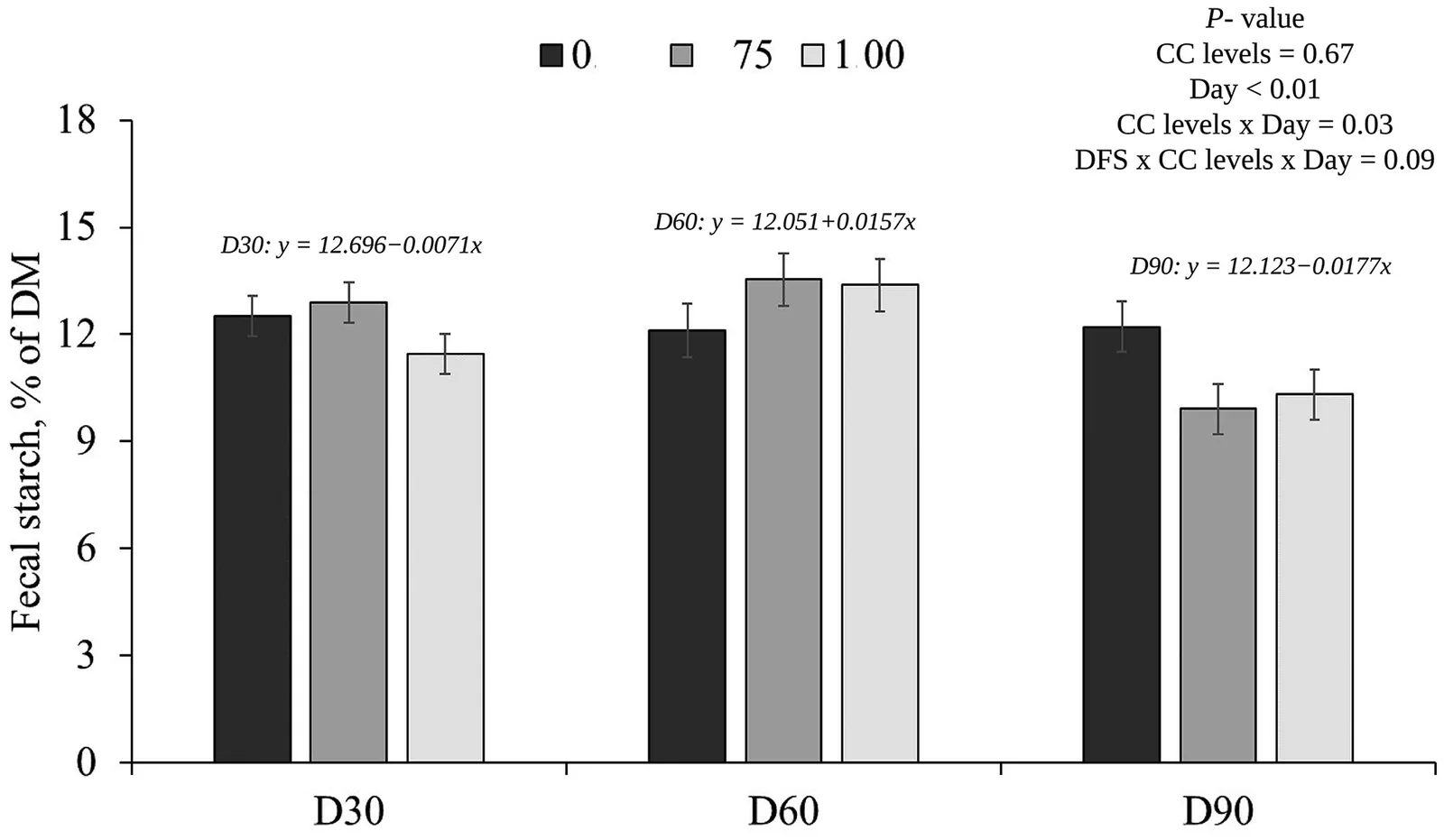
Effect of carbohydrase complex (CC) inclusion levels (0, 75, and 100 mg/kg DM) on fecal starch (% of DM) in crossbred bulls fed diets containing DFS at 45% of DM, evaluated at 30 (D30), 60 (D60), and 90 (D90) d of the finishing period. Error bars represent the standard error of the mean (SEM). there was no effect of CC inclusion levels (*P* = 0.67), whereas day affected fecal starch concentration (*P* < 0.01). An interaction between additive inclusion levels and day was observed (*P* = 0.03), and a tendency for a three-way interaction among DFS, additive inclusion levels, and day was detected (*P* = 0.09). DFS, dry fiber plus solubles; CC, carbohydrase complex.

**Figure 3:**
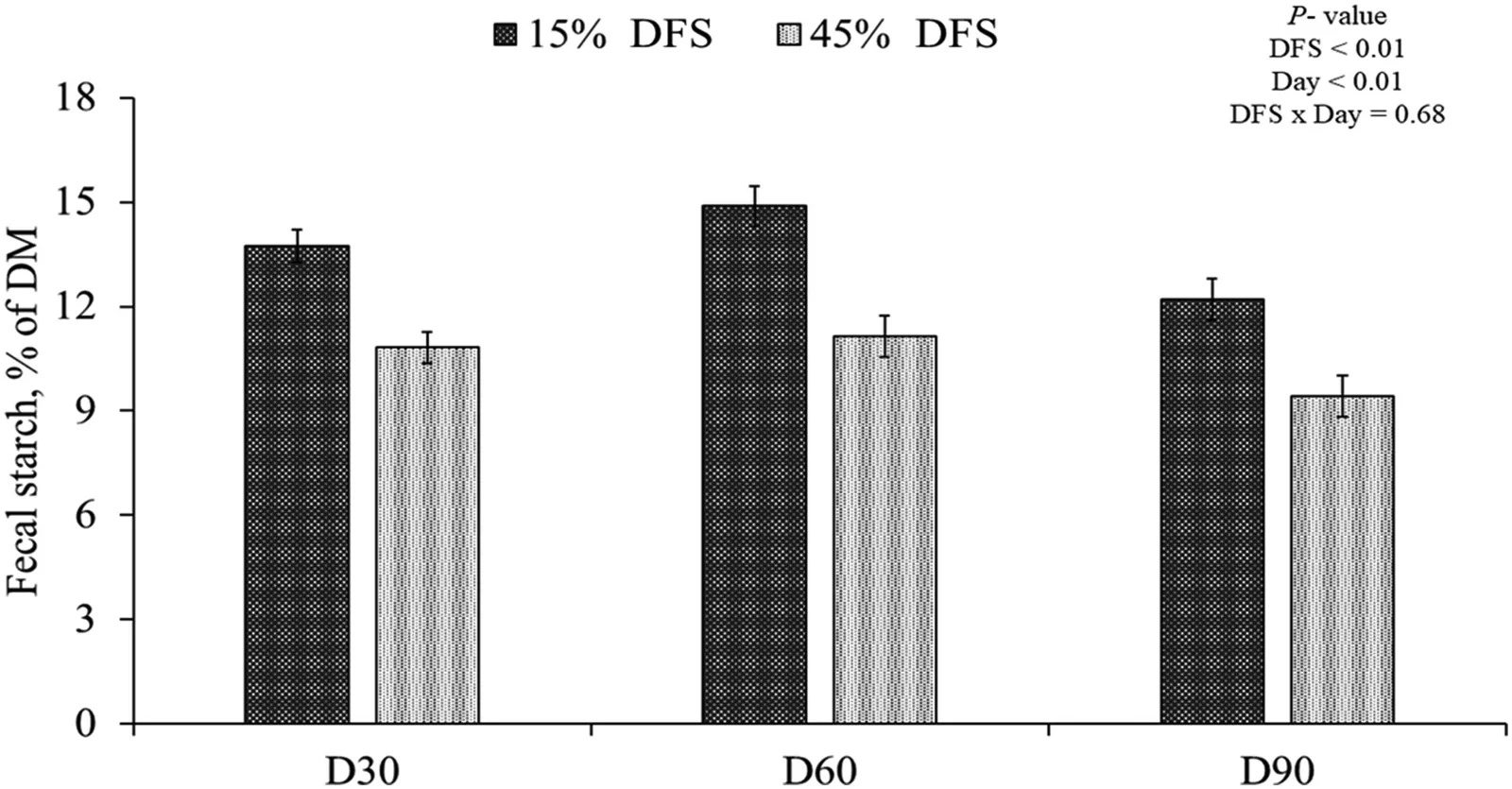
Effect of DFS inclusion level (15 vs. 45% of diet DM) on fecal starch (% of DM) in crossbred bulls, evaluated at 30 (D30), 60 (D60), and 90 (D90) d of the finishing period. Error bars represent the standard error of the mean (SEM). DFS inclusion level (*P* < 0.01) and day (*P* < 0.01) affected fecal starch concentration, whereas no interaction between DFS and day was observed (*P* = 0.68). DFS, dry fiber plus solubles.

No interaction between DFS inclusion and CC levels was observed for energy calculations (*P* ≥ 0.71; Table 6). Inclusion of 45% DFS increased the observed net energy (**NE**) for maintenance (*P* = 0.03) and gain (*P* = 0.04) relative to inclusion of 15% DFS. However, DFS inclusion did not affect the observed: expected NE (*P* ≥ 0.20). CC levels did not affect the energy variables (*P* ≥ 0.83).

**Table 6:** Energy intake of crossbred feedlot bulls receiving high-concentrate diets containing 15 or 45% of DFS and increasing levels of CC (0, 75, and 100 mg/kg of DM).

Items^b^	DFS^a^		CC^a^			SEM	*P*-value		
	15%	45%	0	75	100		DFS	CC	DFS × CC
**NE observed, Mcal/kg**									
** Maintenance**	1.875	1.941	1.898	1.820	1.905	0.04	0.03	0.83	0.72
** Gain**	1.235	1.291	1.254	1.273	1.260	0.03	0.04	0.83	0.75
**NE observed: expected**									
** Maintenance**	0.920	0.936	0.924	0.934	0.926	0.02	0.32	0.83	0.72
** Gain**	0.891	0.816	0.898	0.910	0.901	0.02	0.20	0.88	0.71

a DFS, dry fiber plus solubles; CC, carbohydrase complex. The CC was included at 0 (no inclusion), 75, and 100 mg/kg of diet DM. Effects of DFS, CC, and their interaction (DFS × CC) are presented. Significance was declared at *P* < 0.05.

b NE, net energy.

## Discussion

### DFS (dry fiber plus solubles)

In recent years, several studies have evaluated the inclusion of co-products from the corn ethanol industry in ruminant diets, particularly DDGS and wet distillers grains with solubles (**WDGS**). Results indicate that these ingredients can fully replace conventional protein sources and partially substitute corn in the diet. According to Spowart et al. (2022), both WDGS and DDGS have positive effects on weight gain and feed efficiency of feedlot cattle when included in finishing diets as partial replacements for dry-rolled or high-moisture corn, or combinations of both (Troyer et al. 2023; Sousa et al. 2024). However, it is important to consider the variability among these coproducts, which may differ in fat content (high-fat or de-oiled) (Jolly-Breithaupt et al. 2018; Alhadas et al. 2023) and in the inclusion of solubles (DiCostanzo et al. 2015; Antunes et al. 2025), which affects the inclusion recommendations depending on the responses obtained. In addition, other factors that affect the results would be the protein source replaced by the co-product and the main energy ingredient (eg, dent or flint corn).

The DFS is obtained using a technology called FiberSeparation Technology ([FST] ICM Inc., Colwich, KS), which consists of separating the fibrous fraction of the grain (pericarp) from the other components (germ and endosperm) before the fermentation process, resulting in a co-product with a lower CP content and a higher amount of NDF when compared to the average DDGS composition (NASEM 2016). This difference in composition means that the results regarding the levels of inclusion may differ in relation to the other corn ethanol co-products (Torres et al. 2022).

The literature addressing the metabolism and performance of finishing cattle fed WFS or DFS remains limited. Antunes et al. (2025) did not observe an effect of including 30% DFS in the diet on DMI when compared to diets without inclusion (control). However, Sousa et al. (2024) reported a 12% increase in DMI when cattle were fed dry fiber plus syrup compared with a control diet and a diet containing 40% WDGS, although no differences were observed among DFS, DDGS, and wet fiber plus syrup. In contrast, Lima et al. (2022) observed a linear reduction in DMI with increasing inclusion of WFS in high-energy diets based on ground flint corn and citrus pulp. This response was attributed to physiological mechanisms regulating feed intake in cattle fed diets with high energy density, associated with the increase in dietary NE resulting from WFS inclusion. These contrasting responses may be related to differences in forage sources, the inclusion of citrus pulp in the diet, and the type and processing of corn. In the present study, increasing DFS inclusion from 15 to 45% of diet DM did not affect DMI in either experiment. This may be considered a favorable response, since cattle fed 45% DFS had greater ADG and G:F without increasing feed intake.

Although DMI was not affected, the inclusion of 45% DFS increased NDF and EE intake in Exp. 1. This response can be explained by the greater NDF and EE concentrations of DFS compared with corn, which increased the dietary NDF and EE contents by 60.2% and 4.0%, respectively, relative to the diet containing 15% DFS. In addition, the inclusion of 45% DFS reduced the NFC concentration of the diet by 20.7%; however, NFC and TDN intake were not affected. Although DMI was not statistically influenced by DFS inclusion (*P* = 0.26), the numerical increase of 11.5% in DMI likely contributed to maintaining similar NFC and TDN intakes among treatments.

There are few reports in the literature regarding the effects of DFS on nutrient digestibility. Recent studies have reported reductions in the apparent total-tract digestibility of DM, OM, and EE (Alhadas et al. 2023; Rosa e Silva et al. 2024), as well as CP (Alhadas et al. 2023), when DDG or DDGS were included in feedlot diets for beef cattle. Garland et al. (2019c) reported a reduction in total-tract DM digestibility (79.1 vs. 74.5%) when 40% WFS was included in the diet replacing a blend of dry-rolled and high-moisture corn. Antunes et al. (2025) reported a numerical decrease in digestibility when 30% DFS replaced a blend of ground flint corn, whole cottonseed, and soybean meal. In contrast, Ormond et al. (2025) observed no effect on DM digestibility when DFS was included at 30% of diets containing soybean hulls, ground corn, soybean meal, and cottonseed cake. Similarly, in the present study, the inclusion of 45% DFS did not affect diet DM digestibility compared with the 15% inclusion level. Comparable discrepancies between digestibility and performance responses have been reported in several studies evaluating distillers grains (**DG**), suggesting that improvements in performance may be more closely related to changes in energy partitioning and metabolizable protein supply than to total-tract digestibility.

Regarding ruminal fermentation, Moraes (2023) reported no effect on SCFA with the inclusion of up to 45% DFS in diets containing high concentrate content. Similarly, no effects on ruminal SCFA concentrations were observed with the inclusion of 40% WFS, WDGS, and DDGS in diets for finishing cattle (Garland et al. 2019c). In the present study, the SCFA profile, total SCFA concentration, and the Ac:Prop and AcBut: Prop ratios were not affected by increasing DFS inclusion in the diet from 15 to 45%. In contrast, Antunes et al. (2025) reported that feeding 30% DFS decreased the molar proportion of ruminal acetate and increased ruminal propionate, resulting in a lower Ac:Prop and indicating a more energetically efficient fermentation. The inclusion of DG in cattle diet usually decreases starch, while it increases the NDF content, what should increase rumen Ac:Prop (NASEM 2016; Alhadas et al. 2021).

The increase in the amount of starch consumed is associated with greater production and accumulation of SCFA, resulting in a reduction in ruminal pH. Therefore, the partial replacement of highly fermented carbohydrates, such as starch, by less fermented components, such as fiber, decreases the accumulation of acids, minimizing the risk of a drop in ruminal pH (Klopfenstein et al. 2008; NASEM 2016; Rosa e Silva et al. 2024). In addition, the ruminal pH is also influenced by the diet content of physically effective NDF (**peNDF**), due to its effect on rumination and chewing time (Llonch et al. 2020). The partial replacement of corn by DDGS, DFS, WDGS or WFS decreases starch and NFC, as observed in the present study. However, this change alone is not capable of resulting in an increase in ruminal pH, due primarily to the low peNDF content of corn ethanol coproducts (NASEM 2016). In the Garland et al. (2019c) study the inclusion of 40% WFS replacing part of the dry-rolled: high-moisture corn blend did not affect rumen pH. Antunes et al. (2025) also did not observe an effect on rumen pH when 30% DFS in the diet replaced a blend of ground flint corn, whole cottonseed and soybean meal for finishing cattle, while Moraes (2023) reported a quadratic negative effect on rumen pH when DFS was fed up to 45% of the diet, replacing blends of ground flint corn, whole cottonseed and soybean meal. In the present study, the increase from 15 to 45% of DFS in replacement of ground flint corn decreased the NFC concentration of the diets, however, it did not affect the total NFC intake and increased the ruminal pH, which is a healthier environment considering a diet containing only 8.5% roughage. In the studies of Antunes et al. (2025) and Moraes (2023), DFS replaced blends of ground flint corn, whole cottonseed, and soybean meal. In the present study, increasing DFS inclusion from 15 to 45% primarily replaced ground flint corn, decreasing dietary starch without changing the forage inclusion level.

The use of corn ethanol co-products, such as WDGS and DDGS, typically reduces ruminal ammonia concentration. When accompanied by a reduction in branched-chain fatty acids, this response may indicate decreased ruminal protein degradation (Benchaar et al. 2013), suggesting a greater proportion of rumen undegradable protein (**RUP**). According to NASEM (2016), DDGS contains approximately 67.9% RUP, which is higher than that of most protein ingredients commonly used in feedlot diets and slightly greater than that of corn, the ingredient replaced in the present study. Although specific values for the RUP content of DFS are not available in the literature, it is believed to be similar to that of DDGS. In the present study, increasing DFS inclusion did not affect ruminal ammonia nitrogen concentrations. Antunes et al. (2025) observed a decrease in ruminal ammonia when DFS replaced all the protein supplied by two highly degradable sources, whole cottonseed and soybean meal. However, in the present study, there was no effect of DFS contents on ruminal ammonia, and it can be speculated that this was due to the higher CP content of the 45% diet compared with the 15% (15.5 vs 14.0%, respectively).

Recent literature seems to converge on the effects of the inclusion of DFS on weight gain, with the report that the inclusion of 30 or 45% of DFS increases the ADG of feedlot cattle fed flint corn by 9.5 (Antunes et al. 2025; Moraes, 2023, respectively). When 40% WFS replaced a blend of dry-rolled: high-moisture dent corn, the increase in ADG was 9.35% and 15.4% compared to 0% WFS diets (Garland et al. 2019a, 2019b, respectively). Animals fed 45% DFS had 7.0% greater ADG than those receiving 15% DFS. This response may be explained by the greater energy density of diets with higher DFS inclusion. Feeding 45% DFS increased dietary NEg by 4.53% compared with the 15% DFS diet. Previous work from our laboratory has also reported increased energy density in diets containing DFS (Antunes et al. 2025; Moraes 2023). These results support the concept that corn ethanol coproducts provide greater net energy than the corn-based ingredients they replace, primarily due to their higher fat concentration, greater proportion of digestible fiber, and increased supply of rumen undegradable protein (Klopfenstein et al. 2008; NASEM 2016; Lima et al. 2022).

Because of the greater energy content of DFS compared with ground flint corn, cattle fed 45% DFS had approximately 6.2% greater feed efficiency than those fed 15%. Improvements in the gain-to-feed ratio with increasing inclusion of DG have been consistently reported and are generally attributed to greater dietary NE concentration and an enhanced supply of metabolizable protein, which improve the efficiency of energy utilization for tissue deposition (Klopfenstein et al. 2008; Chen et al. 2024; Ormond et al. 2025).

Carcass responses were generally unaffected by DFS inclusion. HCW did not differ among treatments. The similar carcass weights among treatments are consistent with the comparable DP observed across diets and indicate that carcass deposition largely reflected overall animal growth. As carcass weight is primarily a function of final body weight when DP is similar, the carcass responses observed are consistent with the differences in growth performance associated with dietary energy supply.

Carcass traits are generally not affected by the inclusion of DG in finishing diets. Previous studies have reported that inclusion levels ranging from 0 to 40% did not influence HCW, REA, or marbling (Buckner et al. 2008; Swanson et al. 2014; Lima et al. 2022).

Overall, increasing DFS inclusion from 15 to 45% of dietary DM improved ADG and G:F without affecting intake, digestibility, or carcass characteristics. These results are consistent with both classical and recent literature demonstrating that corn ethanol co-products improve finishing performance primarily through increased dietary energy density and metabolizable protein supply rather than through changes in feed intake or digestibility.

### CC (carbohydrase complex)

The use of fibrolytic enzymes (carbohydrase) in ruminant nutrition aims to act together with the enzymes produced by ruminal microorganisms, increasing fermentative efficiency (Beauchemin et al. 2003; Ramdani et al. 2025), providing soluble carbohydrates from the hydrolysis of the cell wall, and increasing the digestibility of the fibrous fraction (Arriola et al. 2017). The use of these enzymatic complexes can contribute to the total fibrolytic activity in the ruminal fluid (Dias et al. 2022). The CC assists the microbial population in fiber digestion, as exogenous enzymatic activity may reduce the metabolic cost associated with microbial enzyme production and enhance fiber degradation (Beauchemin et al. 2003). Since these enzymes act in the degradation of the plant cell wall, the sugars generated are released (monosaccharides) facilitating the assimilation by microorganisms (Zilio et al. 2019). However, no increase in the digestibility of the carbohydrate fraction of the diet was observed with the use of CC (NDF or NFC).

Enzymes may be resistant to digestion in the rumen and abomasum, as there was an increase in enzymatic activity in the intestine, mainly xylanase activity, indicating that it may affect the utilization of nutrients beyond the animal’s stomachs (Hristov et al. 1998; Ramdani et al. 2025). This has been demonstrated with monogastric animals, where it was observed that these enzymatic complexes act by decreasing the viscosity of the digesta in the duodenum, which also contributes to greater digestion and absorption of nutrients by the animals (Bedford and Cowieson 2020). The current findings demonstrate an increase in EE digestibility with CC inclusion, in addition to a linear increase in CP digestibility with increasing CC levels in diets containing 45% DFS.

Enzymatic complexes for ruminants aim to improve fiber digestion in the rumen through the combined activity of xylanase and cellulase (Holtshausen et al. 2011), and recent studies have focused on the association of these enzymes in multi-enzyme complexes (Ramdani et al. 2025). Xylanases allow greater access of enzymes to cellulose structures (Schaubeder et al. 2022) and can alter the physiological activities of the ruminal microbiota (Colombatto et al. 2003). The inclusion of this enzyme allows an increase in the production of propionate and butyrate, while there is a reduction in the production of acetate and methane (Eun and Beauchemin 2007). In the present study, the inclusion of CC linearly reduced the proportion of ruminal acetate and increased propionate. Although total-tract NDF digestibility was not altered, inclusion of CC may have enhanced microbial access to intracellular carbohydrates via partial cell wall degradation, which may partially explain the shift toward a greater ruminal propionate proportion.

The results of enzyme complexes on ruminal ammonia concentration are still controversial, varying with the type of diet and enzyme dose used. In studies where there was a reduction in the ruminal concentration of ammoniacal nitrogen, this may be related to the increase in NDF and acid detergent fiber (**ADF**) digestibility, and consequently, with the increased use of ruminal ammonia and microbial synthesis (Song et al. 2018; Sakita et al. 2022). The absence of changes in ruminal ammonia nitrogen in this study does not suggest any improvement in microbial growth with CC supplementation.

Data on the effects of CC on animal performance remain inconsistent. While some studies have reported improvements in G:F associated with increased ADG and reduced DMI (Beauchemin et al. 1995), others have observed increased DMI following CC supplementation (Neumann et al. 2018; Fernandes et al. 2024). Despite the positive results, some studies have not observed effects of CC on performance of feedlot cattle (Monteiro et al. 2019). These variations could be attributed to different factors, such as dosage, mode of inclusion (powder or liquid), type of diet, retention time of the diet in the animal’s GI tract, among others (Beauchemin et al. 2003; Ferreira et al. 2025). In the present study, CC was included in powder form (previously homogenized with mineral premix) in a diet containing 8.5% sugarcane bagasse (low quality fiber), with ground flint corn as the main energy ingredient (higher vitreousness than dent corn), and the proposed doses, despite increasing the energy efficiency of ruminal fermentation, did not result in changes in animal performance, as well as in the observed energy density of the diets.

## Conclusion

DFS appears to be a suitable ingredient for inclusion at up to 45% of dietary DM in finishing diets for feedlot cattle based on high proportions of flint corn. Increasing DFS inclusion resulted in greater ruminal pH, ADG, and G:F. With respect to CC, supplementation increased the efficiency of ruminal fermentation, particularly by increasing the molar proportion of propionate and reducing the Ac:Prop ratio. Despite these shifts in fermentation profile, animal performance was not affected, indicating that further research is needed to better understand how this technology can be optimized in feedlot cattle diets.

## List of abbreviations

Ac:Prop: acetate:propionate ratio
AcBut: Prop: (acetate + butyrate): propionate ratio
ADF: acid detergent fiber
ADG: average daily gain
AIC: Akaike information criterion
BFT: backfat thickness
BW: body weight
CC: carbohydrase complex
CP: crude protein
DDGS: dried distillers grains plus solubles
DG: distillers grains
DFS: dry fiber plus solubles
DM: dry matter
DMI: dry matter intake
DP: dressing percentage
EE: ether extract
EG: energy gain
G:F: gain-to-feed ratio (feed efficiency)
HCW: hot carcass weight
ME: metabolizable energy
MEx: maintenance energy expenditure
NDF: neutral detergent fiber
NFC: nonfiber carbohydrates
NE: net energy
NEg: net energy for gain
NEm: net energy for maintenance
OM: organic matter
peNDF: physically effective neutral detergent fiber
REA: ribeye area
RUP: rumen undegradable protein
SCFA: short-chain fatty acids
TCHO: total carbohydrates
TDN: total digestible nutrients
TMR: total mixed ration
WFS: wet fiber plus solubles
WDGS: wet distillers grains plus solubles
